# Axon Biology in ALS: Mechanisms of Axon Degeneration and Prospects for Therapy

**DOI:** 10.1007/s13311-022-01297-6

**Published:** 2022-10-07

**Authors:** Michael P. Coleman

**Affiliations:** John van Geest Centre for Brain Repair, Department of Clinical Neurosciences, University of Cambridge, Robinson Way, Cambridge, CB2 0PY UK

**Keywords:** Axon degeneration, Axonal transport, SARM1, NMNAT2, Programmed axon death, NAD

## Abstract

This review addresses the longstanding debate over whether amyotrophic lateral sclerosis (ALS) is a ‘dying back’ or ‘dying forward’ disorder in the light of new gene identifications and the increased understanding of mechanisms of action for previously identified ALS genes. While the topological pattern of pathology in animal models, and more anecdotally in patients is indeed ‘dying back’, this review discusses how this fits with the fact that many of the major initiating events are thought to occur within the soma. It also discusses how widely varying ALS risk factors, including some impacting axons directly, may combine to drive a common pathway involving TAR DNA binding protein 43 (TDP-43) and neuromuscular junction (NMJ) denervation. The emerging association between sterile alpha and TIR motif-containing 1 (SARM1), a protein so far mostly associated with axon degeneration, and sporadic ALS is another major theme. The strengths and limitations of the current evidence supporting an association are considered, along with ways in which SARM1 could become activated in ALS. The final section addresses SARM1-based therapies along with the prospects for targeting other axonal steps in ALS pathogenesis.

**Supplementary Information:**

The online version contains supplementary material available at 10.1007/s13311-022-01297-6.

## Introduction

A plant with insufficient water wilts from its leaves, but it is the roots that have to be watered. All parts of the plant may eventually be lost if the situation persists, but the leaves are the hardest to maintain and the easiest to sacrifice and regrow. Letting these go first maximizes the chance of overall survival.

One central theme of this review is the extent to which the longstanding debate over ‘dying back’ or ‘dying forward’ models of ALS [1, 2] relates to this simple model of a plant without water. Are axons and their presynaptic terminals lost first in ALS? If so what, if anything, does this mean about whether pathogenesis begins in the soma, the axon or elsewhere, or in different places in different patients?

A second theme, which is closely connected with the first, is the emerging role of SARM1 in ALS. SARM1 is particularly well known for its profound effects in killing axons [3, 4]. Although this prodegenerative role and its regulation in axons was discovered using the experimental platform of axon injury, SARM1 can kill the neuronal soma directly too, for example when it is becomes constitutively active through gain-of-function (GoF) mutation [5, 6], or when it is activated by a toxin [7, 8]. SARM1 also responds to some viruses in ways that are less well understood [9, 10, 11]. What is the evidence so far supporting a role for SARM1 in ALS, and what more do we need to know to confirm this and to understand how widespread its involvement is? And which genetic, environmental and other factors could lead to SARM1 activation in ALS?

The third set of questions relate to whether there is a single mechanism of axon loss in ALS or several. How might the involvement of TDP-43 in most cases relate to axon and synapse loss, and what other risk factors, including ageing, may interact with this to make axons particularly vulnerable?

The final area of focus is therapy. Do we need to target axon survival specifically or might it be sufficient to address underlying issues in other compartments? What treatment or prevention strategies are being pursued and with what success? Could axon regeneration help, and what future options may come available?

## ‘Dying Back’ Pathology in ALS

### What Is ‘Dying Back’ and Does It Occur in ALS?

It is important to clarify what we understand by ‘dying back’ in neurological disorders. The term has several meanings and using them interchangeably can lead to confusion. Each meaning is considered below, along with a critical assessment of whether this applies in ALS.

#### Does Loss of Axons Precede Soma Loss?

The first use of the term ‘dying back’ in toxic neuropathies [12, 13] referred to the loss of axons before the neuronal soma. From this came the notion that death of the soma may sometimes be secondary to loss of axons, caused for example by the lack of retrogradely transported trophic factors.

The extent to which axon loss precedes soma loss in ALS is complicated by the different methods used to assess survival or death in different compartments. The most accurate measure of axon loss in peripheral nerve or ventral roots is electron microscopy, where a dying axon is defined by granular disintegration of the cytoskeleton and mitochondrial swelling [14]. Once the axon fragments [15] and myelin ovoids form, this is considered terminal. Some studies use just neurofilament or myelin staining which, although providing a convenient approximation of axon death, are less sensitive. Some neurofilament epitopes may remain even when ultrastructure is lost and myelin loss is a secondary event [15]. CNS axon survival is sometimes quantified by counting axonal swellings or spheroids [16]. However, it is not completely clear whether these axons are dead as swelling often occurs well before fragmentation [17]. They are, however, unlikely to be functional while swollen. Denervation of neuromuscular junctions (NMJs) is particularly early [18] and this further complicates the assessment.

Neuronal survival on the other hand is assessed using different methods altogether. DAPI or Nissl staining are commonly used to count live neurons, while propidium iodide or TUNEL staining give a measure of neurons that are dying or have been recently lost [19]. Quantifying histopathology, such as TDP-43 aggregation, as a surrogate for cell death introduces even more uncertainty because aggregation does not necessarily mean death [20], even if it is likely these neurons are substantially compromised. Thus, comparing the results of differing measures of axon and soma death, each of varying reliability, does complicate answering this important question.

Nevertheless, from the best estimates using these imperfect comparison methods of the timing of axon and lower motor neuron cell death, it does indeed appear that axons are lost before the cell body, even in structures as proximal as the ventral roots [2]. Moreover, while soma loss in one animal model could be completely prevented by Bax deletion, denervation of NMJs, and symptoms, were only delayed [21]. Of course much of this information is gained from animal models, due to the difficulty of obtaining human tissue in early disease stages. These models vary in how well they represent the human disease and at best often reflect just one of many distinct causes, while most human cases are multifactorial. Some of the best data comes from SOD1 transgenic mice, although the unusual lack of TDP-43 aggregates in SOD1 cases suggests these represent only a small subset of human ALS [22]. Meanwhile, all mouse models have the unavoidable caveats of having shorter axons and shorter lifespans than humans, and consequently often show less severe pathology and symptoms than their direct counterparts in human patients.

#### Are Distal Axon Structures Lost Before More Proximal Regions?

A separate question is whether more distal regions of an axon die before its more proximal regions. This can be very hard to ascertain. There is some evidence for it in some conditions, obtained by measuring axon numbers at different proximal and distal sites relative to those in control nerves, or from longitudinal axon imaging [23]. It is less clear whether this happens in ALS, other than at NMJs. ALS does not show the classical ‘glove and stocking’ pattern of peripheral neuropathies, for example, reflecting length-dependent axon degeneration, and it sometimes shows upper limb onset, where axons are shorter than the lower limbs.

**Fig. 1:**
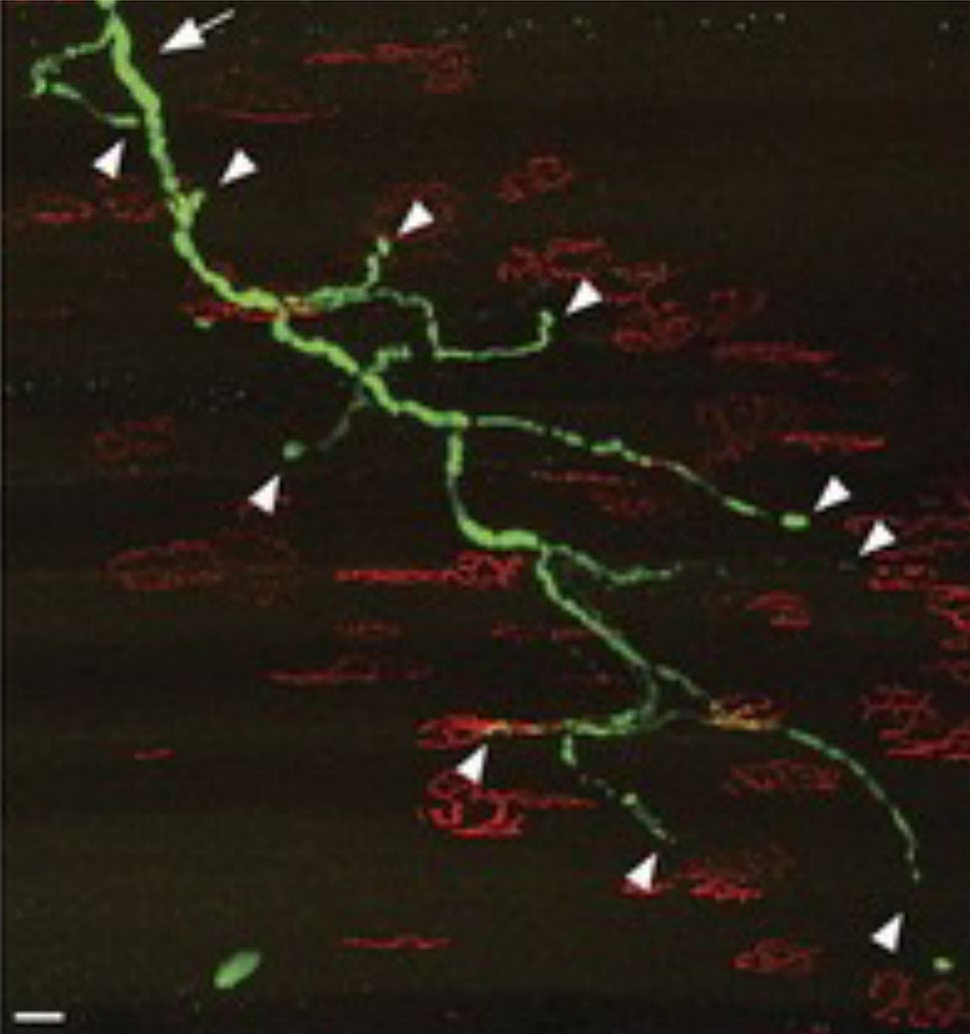
Fully denervated NMJs in SOD1G93A transgenic mice. A complete motor unit in the sternomastoid muscle that lacks a single normal junction. More proximal parts of this intramuscular axon arbor appear substantially normal giving the characteristic ‘winter tree’ appearance. (Reproduced with permission from Schaefer et al. [18])

There is a far clearer picture of the relative timing of terminal synapse loss and the loss of other distal axon structures, especially in NMJs. This is particularly notable in the spectacular ‘winter tree’ images from SOD1^G93A^ transgenic mice with sparsely labelled axons, which demonstrate beyond any doubt that NMJ loss is an early event [18] (Fig. 1). This was also documented in other studies of the G93A and G37R transgenic lines directly comparing the timing of denervation with motor neuron loss. While innervation of fast fatigable muscles is reduced by 40% by P30, motor neuron loss is limited to 20% even at P60 [24, 25]. Denervation and sprouting can occur simultaneously in different parts of the same motor unit [26], and in one human early stage case NMJ denervation also preceded motor neuron loss [2]. Recent studies show similar findings in STMN2 null mice, a protein that regulates microtubule stability and neurite outgrowth [27, 28], whose depletion occurs downstream of TDP-43 ablation [29]. The use of intravital confocal microendoscopy could help gain further data directly in humans and have biomarker potential too [30]. Moreover, peripheral nerve injury studies have repeatedly shown that NMJ loss occurs within less than a third of the time it takes to lose other distal axon structures, both in wild-type animals and those whose axons and NMJs are relatively preserved by *WLD*^S^ [31]. *WLD*^S^ is a mutant gene encoding an NAD-synthesizing fusion protein with an ability to extend the survival of injured axons by tenfold [32]. A similar situation arises in the absence of nerve injury, when *Nmnat2*^−/−^ mice are rescued by *WLD*^S^, with NMJ denervation causing paralysis between 7 and 10 months of age [33]. It is important to remember, however, that NMJ loss does not necessarily reflect the state of the underlying axon trunk (Fig. 1), and that reinnervation of vacant endplates by sprouting from surviving axons complicates this analysis.

#### Does Axon Loss Cause Secondary Death of the Motor Neuron Soma?

The extent to which axon loss leads to neuronal death as a secondary event depends on the neuron type and developmental stage, since some neurons are more sensitive to losing their axon than others. Nerve injury studies show that adult motor and sensory neurons in lumbar spinal cord both survive loss of their distal axons, which is important because neuron survival is an essential prerequisite for peripheral nerve regeneration. However, if adult motor axons are broken at a much more proximal site, for example by ventral root avulsion, this does cause apoptotic death of the adult lower motor neuron soma [34]. In neonates, this occurs even if the injury is further distal, for example in sciatic nerve, perhaps reflecting a greater dependence on retrograde survival signals at this critical stage of development when neurons are competing with one another to innervate their targets.

The effect of axon injury within the CNS on neuron survival is harder to assess because of the practical challenges of CNS axon lesion. However, there is clearly a mixture of responses from different neuron types. Whereas some neuron types show the typical chromatolytic response to axon injury but survive [35], retinal ganglion cell neurons are one example of a CNS neuron population that does undergo apoptosis following optic nerve lesion [36]. Current understanding of upper motor neuron survival after spinal cord injury is that most do survive without an intact axon [37].

Some specific motor neuron disease models in mice show what could be interpreted as motor neuron loss secondary to axon loss. For example, the progressive motor neuronopathy (*pmn*) mouse, which undergoes massive motor axon loss in the first weeks after weaning due to deficiency of a key protein for microtubule formation and axonal transport [38], has motor neuron loss that follows slightly after axon loss [39]. However, loss of each compartment is temporarily but strikingly rescued by the Wallerian degeneration slow (*WLD*^S^) gene [39]. As *WLD*^S^ has been shown to rescue only axons directly, not the soma [34], this together with the temporal sequence strongly suggests that loss of the soma in *pmn* mice is secondary to axon loss. This in turn likely reflects the early developmental stage when the soma is more dependent on retrograde signals for survival, as these cannot be delivered if axons are lost. While *pmn* is not itself a model for any known form of ALS, the human ortholog of the affected gene is biallelically mutated in a rare form of distal spinal muscular atrophy with encephalitis [40], so it has been associated with motor neuron loss in humans too.

### Does This Mean ALS Pathogenesis Arises Within Axons?

The short answer to this question is ‘not necessarily’ but we need to examine currently known ALS risk factors and ask whether it may be the case, or at least partially the case, in some patients.

It is highly likely that many of the early steps in pathogenesis take place within the soma. Not only are there very early ultrastructural changes in the motor neuron soma in animal models [41] but there are increasingly well-understood deleterious consequences of several major causes of familial ALS that operate within the soma. Pathogenic mechanisms such as disruption of nuclear-cytoplasmic transport and mRNA processing, stability and transport reflect events that take place largely or exclusively in the soma. For example, the neurotoxicity of C9orf72 hexanucleotide repeat expansions, the most common known cause of familial ALS and a common risk factor for sporadic ALS, can be potently suppressed in *Drosophila* by RanGAP, a regulator of nuclear-cytoplasmic transport [42], and there is additional support for such a mechanism in hiPSC-derived motor neurons from patients. Causative mutations in TDP-43 in many familial ALS cases, a protein whose aggregation also occurs in nearly all familial and sporadic cases, also alter its nuclear-cytoplasmic distribution and the processing and distribution of many associated RNAs [43] much of which occurs in the cell soma.

However, even causative events with such compelling evidence of effects within the soma can also have consequences within the axon itself that may not be immediately obvious, so it is important we do not presuppose which compartment mediate/s the disease process. For example, C9orf72-derived arginine-containing dipeptide repeats, which are associated with its ALS mutation and remain a good candidate for the pathogenic mechanism, disrupt axonal transport [44], while TDP-43 also has a role in regulating axonal protein synthesis [45] and it pathogenic variants also disrupt axonal transport of signalling endosomes [46]. Additionally, premature termination of the transcript for axonal protein STMN2, downstream of TDP-43 disruption, lowers axon outgrowth [47, 48]. Nevertheless, it is remarkably difficult to be sure whether even these events occur within the axon or whether they are secondary to changes in the soma, such as mitochondrial bioenergetic deficits [49].

The nature of several other genetic risk factors for ALS appears to link them more specifically to axons, and to axonal transport or cytoskeleton in particular. For example, pathogenic variants of the anterograde and retrograde motors or associated proteins KIF5A and dynactin subunit 1 (DCTN1; p150^Glued^) both disrupt axonal transport [50, 51], and ALS-associated variants of the heavily phosphorylated repeat region of the heavy neurofilament subunit (NEFH) are likely to cause axonal swellings [52]. But these are rare risk factors, and even here it is hard to be absolutely sure of an axonal site of action because every axonal protein ultimately has to traffic through the soma to get there, and often they have soma functions too.

**Fig. 2:**
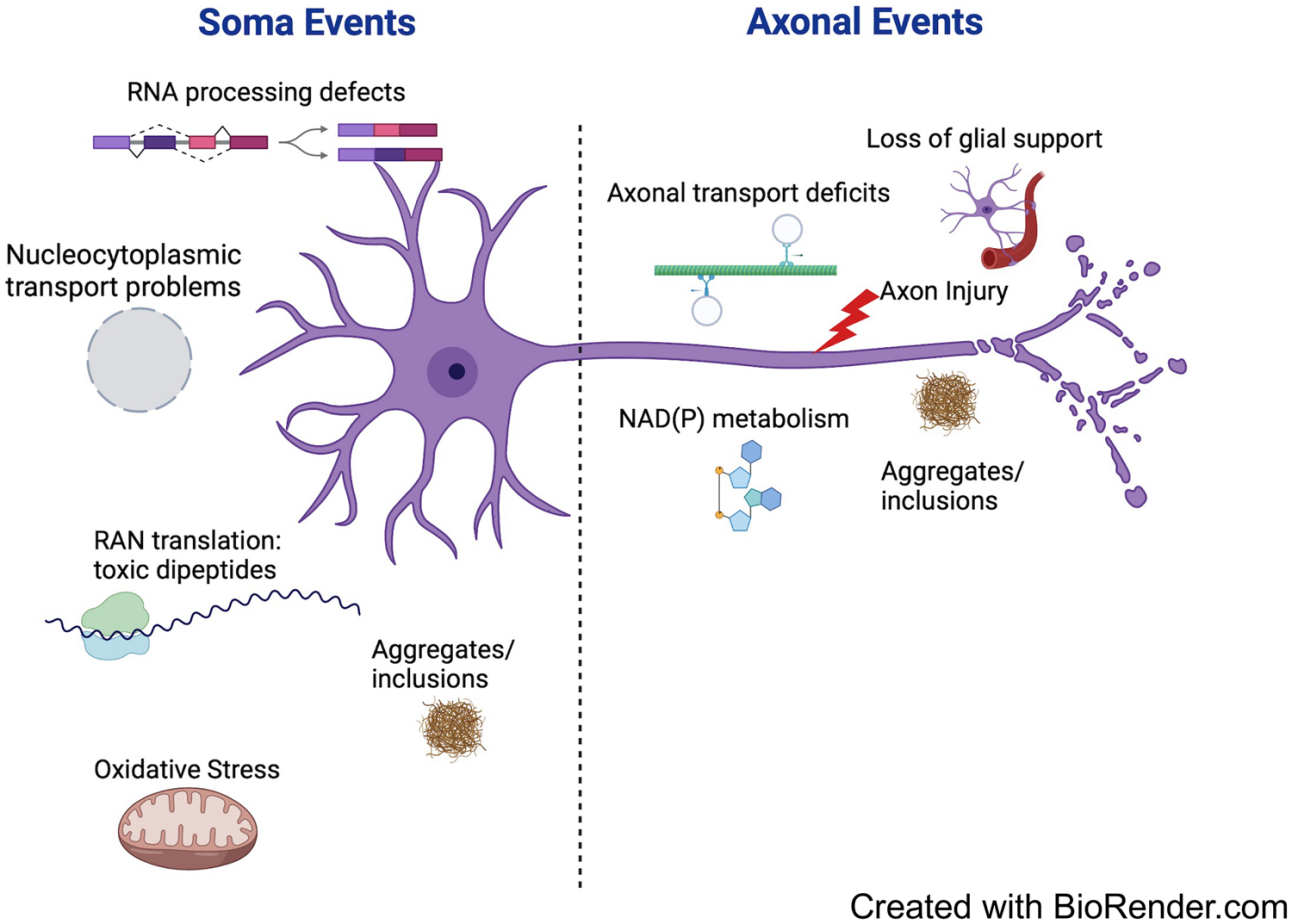
Soma and axonal deficits in ALS. Many of the most important causal steps in ALS are likely to take place in the soma but some are primarily axonal. ‘Dying back’ can result from a failure of the weakened soma to support its axon but this may be particularly reinforced when combined with additional problems in the axon

Finally, axons are known to be highly vulnerable to proposed environmental risk factors for sporadic ALS, such as traumatic brain injury [53]. Axonal transport also declines greatly with age [54], the biggest single risk factor in ALS, and loss of the glial glutamate transporter GLT1 in ALS [55] appears likely to impact axons more than the soma. Thus, in summary, it seems highly likely that axonal events do contribute directly to pathogenesis in some cases. However, given the specificity of the disorder for motor neurons as well as the evidence of disruption of soma events, it is likely that such events often weaken the soma’s ability to support the axon. This may prime the axon for degeneration, in ways that manifest when axons are also directly disrupted in some way, whether by mutation of an important axonal protein, injury or a failure of glial support (Fig. 2).

## SARM1 and ALS

### SARM1 Actions

**Fig. 3:**
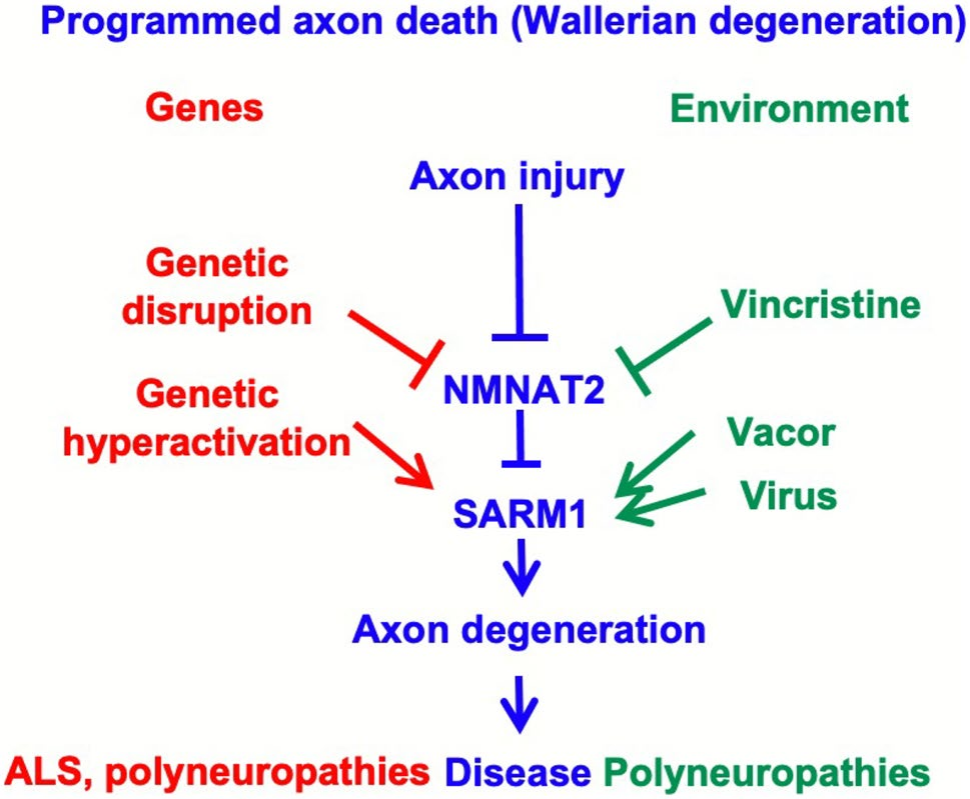
The multiple triggers of programmed axon death in human disease. The NAD(P)ase and/or base exchange activity of SARM1 drives degeneration. It occurs in axons specifically when its upstream regulator, NMNAT2, falls below a threshold level, which may occur after axon injury, NMNAT2 LoF mutation or axonal transport deficits, such as caused by some cancer chemotherapeutics targeting microtubules. SARM1 can also be activated directly by GoF mutation or some toxins, and this can also cause death of the soma. Some viruses also cause SARM1-dependent degeneration

SARM1 is an enzyme and TLR adapter protein driving the central execution step of programmed axon death (Fig. 3) [3, 56]. It has multiple enzyme activities, including both the degradation and cyclisation of NAD and NADP, and base exchange activity that switches the nicotinamide of either coenzyme for another pyridine base [57, 58]. This base exchange function can become dominant over the others in some circumstances [59]. Depletion of NAD, and consequently of ATP, has been widely assumed to be the proximal cause of SARM1-dependent death, but there is so far no firm evidence as to whether this or changes in one of its other substrates or products is causative. Removal of one SARM1 product, cADPR, was not found to be protective, but the roles of other calcium modulating signals remain untested, including NaADP [59] and ADPR [60]. Loss of ROS buffering capacity is another likely consequence of SARM1 activation, reflecting NADP(H) loss and full spectrum of base exchange products remains unknown. It will be important to remain open minded until this is resolved.

### SARM1 Regulation

SARM1 has a low level of basal activity but all of its enzyme activities are strongly activated by nicotinamide mononucleotide (NMN), the substrate of its upstream regulator, NMNAT [58, 59]. High levels of NAD oppose this activation by binding to the same site on its N-terminal inhibitory domain [59, 61, 62]. Thus, loss of the main axonal NMNAT isoform, NMNAT2, a protein absolutely required for axon survival [63, 64] appears to be what provides the axonal specificity of degeneration after axon injury, or in axonal transport disorders [65, 66], or when NMNAT2 is genetically disrupted [64, 67, 68, 69], since NMNAT1 supplies the same activity in the soma. Most strikingly, the perinatal lethality and axon growth deficit in *Nmnat2*^−/−^ mice are completely rescued by the simultaneous removal of SARM1 [33] indicating the extremely strong therapeutic potential of this drug target.

While SARM1 activation due to NMNAT2 loss appears to be axon-specific, there are at least three ways it can be activated in, and kill, the neuronal soma too. First, mutation of NMNAT1 in Leber’s congenital amaurosis type 9 (LCA9) causes retinal ganglion cell death [70]. Findings made using an *Nmnat1*^−/−^ mouse model suggest this mechanism is SARM1-dependent [71]. Thus, just as removal of the primary axonal NMNAT (isoform 2) kills axons, removal of NMNAT1, a nuclear protein [32], kills this type of neuron. However, there is no evidence of any effect of NMNAT1 disruption on motor neurons.

Second, SARM1 can be directly activated by toxic metabolites of several nicotinamide analogues [7, 8], and this can occur in the soma just as readily as in axons. This was first discovered by understanding the toxic action of the disused rodenticide vacor, which is metabolised by NAMPT to vacor mononucleotide (VMN), an NMN analogue that binds to the same site on the SARM1 inhibitory N-terminal ARM domain as NMN and activates SARM1 NADase activity even more potently [7]. A similar action by 3-acetyl pyridine [8] raises the important question of whether other pyridines still in our environment today could drive SARM1-dependent neuronal and axonal death. Athough vacor exposure in humans was associated with polyneuropathy [72] rather than ALS, it can clearly kill other neuron types [7] so it is plausible that environmental activators of SARM1 could combine with other ALS risk factors to contribute to ALS.

The SARM1 activation mechanism most directly related to ALS is its direct hyperactivation by rare genetic variants that disrupt its N-terminal, inhibitory ARM domain. Mutations within this region that strongly enhance basal SARM1 NADase activity are associated with sporadic ALS and they are sufficient to enhance neuronal vulnerability to other stresses [5, 6]. This association is highly significant at the single gene level, indeed known GoF variants were unique to patients among over 11,000 patients and more than 10,000 controls [5]. Because they are so rare, their association with ALS does not pass the threshold for genome-wide significance at present, but independent of this finding, there is genome-wide association of a more common intragenic SNP within the *SARM1* gene with sporadic ALS [73]. The genomic distance between the GoF coding variants and the lead GWAS SNP is only 8–11 kbp, so while it is unlikely that these very rare variants contribute much to the GWAS signal themselves, the combination of these independent findings strongly suggests a wider role for SARM1 in ALS. One unifying model would be that SARM1 can be activated by more than one mechanism to contribute to sporadic ALS, and that higher levels of SARM1 gene expression make axons more vulnerable to such effects.

Finally, the recent finding that zika virus causes SARM1-dependent neuronal death [9], along with earlier indications of similar effects with both rabies and West Nile virus [10, 11], albeit so far by unknown mechanisms, raise the important question of whether endemic viruses could make an as-yet unrecognised contribution to neurodegenerative disorders such as ALS by acting on SARM1. At present, this can be only speculative, but since an environmental component in sporadic ALS of around 40% needs to be accounted for [74], and since some viruses including rabies and zika have indeed been associated with ALS risk and motor neuron death [75, 76, 77], it will be important to consider.

## Mechanisms of Axon Loss in ALS

### One Mechanism, Several or Many?

ALS results from varying combinations of a large number of risk factors. Twin studies show sporadic ALS has up to 61% heritability with the remaining ca. 39% presumably reflecting a range of environmental risk factors [74]. With up to 50 causative genes already identified from familial cases and more remaining to be identified, it clear that the genetics alone involves multiple mechanisms. Some of these also influence risk of sporadic ALS. Much less is known about environmental risks but some that have been proposed based on epidemiology studies are environmental toxins [78], strenuous exercise [79] and traumatic brain injury (TBI) [53], although better preclinical models are required to establish causation rather than reverse causation for TBI [80]. Ageing is also the greatest single risk factor.

This wide range of genetic and environmental risk factors [81] suggests the specific combination that causes pathogenesis could differ considerably from one patient to the next. However, the near-ubiquitous presence of TDP-43 aggregates [82] suggests convergence of these varying initial causes onto one or a few central mechanisms. In this context, it is important to ask whether the cause of axon loss in ALS has one, several or many mechanisms. It is likely to be some time before many environmental risk factors in particular are fully understood because of the many difficulties in identifying them, although there are a number of good candidates and the use of Mendelian randomization promises to help in establishing a causative role [81].

**Fig. 4:**
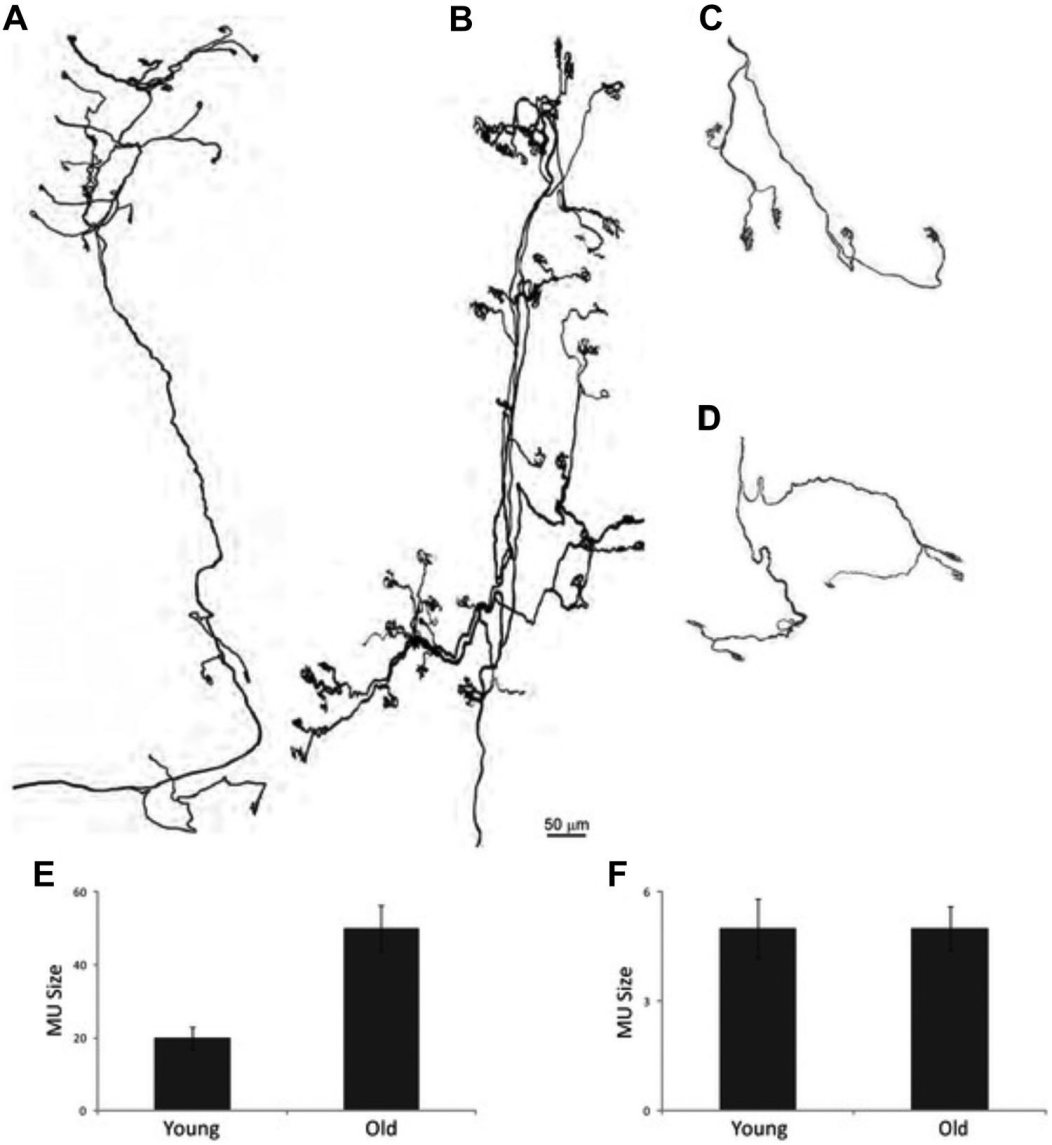
Age-related changes in motor unit size. **A**–**D**: Motor units from the omohyoid (**A**, **B**) and extraocular (**C**, **D**) muscles of young adult (**A**, **C**) and old (**B**, **D**) mice. **E**, **F**: Motor unit size in young adult and old omohyoid (**E**) and extraocular (**F**) muscles showing the increase in size specifically in the omohyoid motor units due to sprouting of surviving motor neurons. (Reproduced from Valdez et al. [83])

The model in Fig. 2 proposes that ‘dying back’ can occur when a compromised motor neuron soma fails to support its large axon, and in particular its terminal arbor. Motor neuron axons are already orders of magnitude larger than the soma that supports them, but in some muscles, they also more than double in size during normal ageing [83]. This occurs because some axons are lost altogether and their surviving neighbors sprout to innervate the vacated postsynaptic targets [83] (Fig. 4). In ALS, there is additional loss of axons beyond this usual age-related change, especially of fast, fatiguable axons [84]. Thus, the surviving slower motor units, which sprout to compensate for these losses, may eventually overreach themselves. The decline in axonal transport during normal ageing [54], which is sometimes also exacerbated in ALS [46], is likely to make these expanded arbors even harder to maintain as the disease progresses. This could help to explain why ageing is the biggest single risk factor.

In other circumstances when axons lack sufficient resources from the soma, it is often the supply of the labile, but essential axonal protein NMNAT2 that limits their survival [56, 63]. This was revealed using studies of axon transection [63] but it is also the case when axonal transport is impaired [39], protein synthesis is blocked in the soma [63] and when RNA metabolism is disrupted by TDP-43 mutation [85], since blocking the same pathway promotes axon survival in each case. Other axonal proteins or other factors may become limiting in time, but it is often the shortage of NMNAT2, leading to activation of SARM1, that is the proximal cause of axon death due to the short half-life and essential nature of NMNAT2 [63].

The extent to which this pathway contributes to ALS remains unknown but GWAS association with the SARM1 chromosomal locus [73] indicates that one or more genes in that region makes a widespread contribution, and a SNP influencing the expression level of SARM1 could determine how strongly the axon responds to compromised support by the soma. However, although many mechanisms converge on programmed axon death (Fig. 3) [86], it may not be the only death signal that arises from cell body impairment. For example, induction of apoptosis in the soma of intact neurons causes caspase-dependent death to spread from the soma to the axon even though caspases do not seem to be involved in programmed axon death [87]. Moreover, SOD1 transgenic mouse models of ALS show little or no improvement when programmed axon death is blocked, either at the NMNAT (WLD^S^) level or the SARM1 level [88, 89] despite some evidence that the soma is impaired very early in these models [41]. NMJ denervation following STMN2 depletion is also independent of SARM1 [29], and glial-derived toxicity, or loss of glial support are further mechanisms that may contribute [90].

### Axon-Intrinsic Events

Part 1(b) introduced the concept that even if many of the primary pathogenic steps in ALS occur in the soma, some patients have risk factors that strongly suggest axon-intrinsic mechanisms. These are discussed here in more detail. According to the model proposed in Fig. 2, these axon-intrinsic events may tip the balance towards disease when the soma is weakened, or they may modify age-of-onset.

We have previously proposed that there is a spectrum of intrinsic axon vulnerability in the human population, based on expression and activity of SARM1 and its regulator NMNAT2 [56]. This is supported by the identification of naturally occurring harmful alleles (NMNAT2 LoF, SARM1 GoF) and protective alleles (SARM1 LoF and dominant negative) [5, 67, 68, 91], and by the wide range of NMNAT2 expression in humans [92]. It is unknown whether such a spectrum influences the likelihood of axon degeneration in ALS although animal model data [93] support its involvement in many disorders of long axons including some forms of ALS and other motor neuron disorders [39, 85]. However, while SARM1 GoF alleles are strongly associated with ALS at the individual gene level [5], it is also clear that these drive soma death as well as axon death. Thus, it is NMNAT2 that brings axon specificity to the programmed axon death mechanism, not SARM1, and any role NMNAT2 may play in ALS remains unexplored. At present, the consequences of the very rare NMNAT2 LoF coding variants in humans are limited to polyneuropathies [67, 68].

Among the other most convincing axon-intrinsic mechanisms are the many causes of cytoskeletal, and particularly axonal transport dysfunction [94]. Among these, the anterograde axonal transport motor KIF5A stands out as a risk factor for the survival of long axons in multiple disorders, including hereditary spastic paraplegia (HSP) [95], Charcot-Marie-Tooth (CMT) disease Type 2 [96], adult onset distal SMA [97], and possibly neonatal intractable myoclonus [98], although whether this last one involves axon loss is less clear. Intriguingly, there may be a degree of domain specificity regarding which KIF5A variant contributes to which disorder, with motor domain variants predominantly linked to HSP and CMT2 and tail (cargo-binding) domain variants to ALS and neonatal intractable myoclonus, although there are exceptions [99]. Nevertheless, the phenotype of the null mice, and their neurons in primary culture [100] clearly indicate that KIF5A is essential for both axonal transport and the ability to sustain long axons. Taken together with evidence of KIF5A functional alteration by ALS-specific variants [50], it seems highly likely that patients with KIF5A variants activate axon-intrinsic mechanisms contributing to the disorder.

While axonal transport defects may contribute to disease in KIF5A cases, and potentially in others influencing cytoskeleton or motor proteins such as dynactin subunit 1 (DCTN1), neurofilament proteins (NF-L, NF-H), spastin (SPAST) and tubulin alpha 4a (TUBA4A) [94], the wider role of axonal transport deficits in ALS remains unknown. This is in part because it is hard to exclude the possibility that axonal transport is impaired secondarily to other events, including impairment of a cell body no longer able to supply everything required for transport, such as motor proteins, components of microtubules and mitochondrial or glycolytic proteins needed to generate sufficient ATP. Indeed, while early deficits of axonal transport have been reported in mouse SOD1 models [101], other studies have separated these from the causative steps in disease [102, 103]. Moreover, while mutant TDP-43 has been found to cause defects in axonal transport of signalling endosomes, mutant FUS does not [46].

Local protein synthesis in axons is another vital mechanism that may be disrupted in ALS by an axon-intrinsic mechanism. One reflection of this could be that the appearance of aggregates of RNA binding protein TDP-43 in axons precedes their degeneration in patients [104]. TDP-43 has a role in regulating local translation in axons [45] that influences the axonal transcriptome [105] and appears to have pathogenic potential [106]. The early denervation of NMJs in mice lacking TDP-43 target STMN2 [29] could represent one mechanism by which TDP-43 influences the survival of axonal compartments directly.

## Targeting Axons for Therapy

In view of the above discussion of whether ALS pathogenesis is driven by soma and/or intrinsic axonal events, and how the balance may differ between different patients according to the presence or absence of axon-specific risk factors, it is important to ask whether axons need to be targeted directly for an effective therapy, or whether it is sufficient to remove primary causes that may lie elsewhere.

Axon-based strategies are likely to play important roles in combinatorial therapies for ALS but their potential for use in isolation may be limited to patients where the predominant risk factor is an axon-intrinsic one. For example, when an axonal role of KIF5A or TDP-43, or a deficit in local translation, makes a substantial contribution, targeting these consequences in axons could be particularly useful. Possible methods include boosting fast axonal transport by inhibition of p38 MAPK [107], a strategy that has been effective in animal models [108], or by HDAC inhibition [109], or alternatively miRNA or ASO based strategies to influence local translation [110]. An ASO-based correction of the STMN2 premature termination defect downstream of TDP-43 [47, 48] could be one example, and such trials are in progress. Intramuscular delivery of viral vectors for gene therapy could be an effective way to deliver such therapies to distal motor axons, in addition to delivering therapies to the soma via retrograde axonal transport [111].

In cases where a weakened soma fails to support the large, and potentially expanded axonal arbor (Fig. 2), leading to programmed axon death if insufficient NMNAT2 is delivered, a SARM1-blocking therapy could be effective. Methods under investigation include inhibition [112], ASOs [113] or disruption of the SARM1 activation mechanism [61]. Importantly, the use of axon transection studies to elucidate much of the NMNAT2-SARM1 mechanism may have created an impression that blocking SARM1 can only protect axons for a few weeks. This is true after transection [3] but not when there is a specific shortage of NMNAT2 [33] or direct SARM1 activation by a toxin [7]. In such cases, removing SARM1 provides long-term, and even lifelong protection. The small minority of patients with SARM1 GoF variants [5] are also promising potential recipients for SARM1 blocking therapies, even if the harmful effect of constitutively-active SARM1 in these patients may also influence the soma.

In connection with this, there is now a substantial literature and wider public discussion of therapy and prevention of neurological disorders using precursors of NAD, such as nicotinamide (Nam) and its riboside (NR) or mononucleotide (NMN) [114, 115, 116]. These do seem able to boost NAD to varying degrees, to protect neurons and axons under some circumstances, and even to show so promising preliminary signs in clinical trials for some disorders [115]. However, for NMN, there are important questions to answer regarding how much of this charged molecule actually gets into cells. All of them, however, come with one very important caveat: their ability to raise intracellular or intra-axonal NAD depends on the availability of sufficient NMNAT to convert NMN into NAD. When there is insufficient NMNAT, as in the axons of an individual with an NMNAT2 LoF variant [67, 68], and perhaps in distal axons in ageing or in a recipient of vincristine for cancer chemotherapy [65], this could instead drive the accumulation of NMN. NMN is an activator of SARM1 NADase [58, 59, 61], so its accumulation could drastically lower NAD, exactly the opposite of the intended therapeutic action and likely to lead to a harmful outcome. Thus, while an appropriate NAD precursor could be beneficial in some patients, it has the potential to be quite harmful in others. More studies are required to establish who is likely to benefit from such an approach and who is not.

Finally, there are many proposed strategies to boost compensatory sprouting [117, 118] or longer-range axon regeneration [119]. These could bring substantial benefits in the early to middle stages of disease as postsynaptic sites vacated by degenerating vulnerable fibers are reinnervated by surviving motor units. One key consideration, however, is whether these expanded arbors overreach themselves, and especially whether they can be supported into old age when NMNAT2 axonal transport declines [54]. Thus, methods to enhance regeneration and sprouting could be at their most effective if combined with methods to raise NMNAT2 levels, or block SARM1 in distal axons, enabling these expanded arbors to be retained as an individual ages.

## Summary and Perspectives

In summary, there are multiple risk factors for ALS, some acting in the soma, some in axons and some potentially in glia or elsewhere. These are all likely to contribute to pathogenesis in varying degrees in different patients. However, there are common features that suggest convergence in most cases onto a mechanism involving TDP-43, probably reflected in its near ubiquitous aggregation, and ‘dying back’ of axons particularly as defined by NMJ denervation. The emerging links to SARM1, including the GWAS association of its chromosomal locus [73], suggest there could be a common SARM1-dependent mechanism. STMN2 depletion also appears to be a common mechanism influencing NMJ innervation, although perhaps not in a SARM1-dependent manner [29]. Thus, addressing these two, potentially independent contributors to axon and NMJ survival, are promising directions for axonal therapies, and they could be especially effective in combination with methods to increase compensatory sprouting.

## Supplementary Information

Below is the link to the electronic supplementary material.

Supplementary file1 (PDF 491 KB)

Supplementary file2 (PDF 109 KB)
