# Supplementary material for: Axon Biology in ALS: Mechanisms of Axon Degeneration and Prospects for Therapy
Source: Neurotherapeutics. 2022 Oct 7;19(4):1133–44. doi: 10.1007/s13311-022-01297-6 (PMC9587191; doi:10.1007/s13311-022-01297-6)
Supplement: Supplementary file 1 — Supplementary file1 (PDF 491 KB) [file 13311_2022_1297_MOESM1_ESM.pdf]

Please wait...

If this message is not eventually replaced by the proper contents of the document, your PDF viewer may not be able to display this type of document.

You can upgrade to the latest version of Adobe Reader for Windows®, Mac, or Linux® by visiting http://www.adobe.com/products/acrobat/readstep2.html.

For more assistance with Adobe Reader visit http://www.adobe.com/support/products/acrreader.html.

Windows is either a registered trademark or a trademark of Microsoft Corporation in the United States and/or other countries. Mac is a trademark of Apple Inc., registered in the United States and other countries. Linux is the registered trademark of Linus Torvalds in the U.S. and other countries.

http://www.springer.com/journal/13311

Neurotherapeutics
The Journal of the American Society for Experimental Neurotherapeutics
Editor-in-Chief: Mouradian, M.
ISSN: 1933-7213 (print version)
ISSN: 1878-7479 (electronic version)
Journal no. 13311
