# Supplementary material for: Axon Biology in ALS: Mechanisms of Axon Degeneration and Prospects for Therapy
Source: Neurotherapeutics. 2022 Oct 7;19(4):1133–44. doi: 10.1007/s13311-022-01297-6 (PMC9587191; doi:10.1007/s13311-022-01297-6)
Supplement: Supplementary file 2 — Supplementary file2 (PDF 109 KB) [file 13311_2022_1297_MOESM2_ESM.pdf]

CCC | RightsLink

? Help | Live Chat | Michael Coleman

A compensatory subpopulation of motor neurons in a mouse model of amyotrophic lateral sclerosis
Author: Jeff W. Lichtman, Joshua R. Sanes, Anneliese M. Schaefer
Publication: Journal of Comparative Neurology
Publisher: John Wiley and Sons
Date: Aug 4, 2005

Copyright © 2005 Wiley-Liss, Inc.

Order Completed

Thank you for your order.

This Agreement between Michael P Coleman ("You") and John Wiley and Sons ("John Wiley and Sons") consists of your order details and the terms and conditions provided by John Wiley and Sons and Copyright Clearance Center.

**License number**	Reference confirmation email for license number
**License date**	Sep, 01 2022

Licensed Content

**Licensed Content Publisher**	John Wiley and Sons
**Licensed Content Publication**	Journal of Comparative Neurology
**Licensed Content Title**	A compensatory subpopulation of motor neurons in a mouse model of amyotrophic lateral sclerosis
**Licensed Content Author**	Jeff W. Lichtman, Joshua R. Sanes, Anneliese M. Schaefer
**Licensed Content Date**	Aug 4, 2005
**Licensed Content Volume**	490
**Licensed Content Issue**	3
**Licensed Content Pages**	11

Order Details

**Type of use**	Journal/Magazine
**Requestor type**	Publisher (STM Signatory)
**STM publisher name**	Springer Nature
**Is the reuse sponsored by or associated with a pharmaceutical or medical products company?**	no
**Format**	Print and electronic
**Portion**	Figure/table
**Number of figures/tables**	1
**Will you be translating?**	No
**Circulation**	1000 - 1999

About Your Work

**Title of new article**	Axon Biology in ALS: Mechanisms of axon degeneration and prospects for therapy
**Lead author**	Michael Coleman
**Title of targeted journal**	Neurotherapeutics
**Publisher**	Springer Nature
**Expected publication date**	Oct 2022

Additional Data

**Portions**	Figure 5b

Requestor Location

**Requestor Location**	Michael P Coleman<br>The Babraham Institute<br>Babraham<br><br>Cambridge, other CB22 3AT<br>United Kingdom<br>Attn: Michael P Coleman

Tax Details

**Publisher Tax ID**	EU826007151
**Customer VAT ID**	GB823847609

Billing Information

**Billing Type**	Invoice
**Billing address**	Michael P Coleman<br>John van Geest Centre for Brain Repair<br>Robinson Way<br>Cambridge<br>Cambridge, United Kingdom CB2 0PY<br>Attn: Michael P Coleman

Price

**Total**	0.00 GBP

Would you like to purchase the full text of this article? If so, please continue on to the content ordering system located here: Purchase PDF
If you click on the buttons below or close this window, you will not be able to return to the content ordering system.

Total: 0.00 GBP

CLOSE WINDOW

© 2022 Copyright - All Rights Reserved | Copyright Clearance Center, Inc. | Privacy statement | Data Security and Privacy | For California Residents | Terms and Conditions
Comments? We would like to hear from you. E-mail us at customercare@copyright.com
